# Time-Dependent Antimicrobial Activity of Filtering Nonwovens with Gemini Surfactant-Based Biocides

**DOI:** 10.3390/molecules22101620

**Published:** 2017-09-27

**Authors:** Katarzyna Majchrzycka, Małgorzata Okrasa, Justyna Szulc, Bogumił Brycki, Beata Gutarowska

**Affiliations:** 1 Department of Personal Protective Equipment, Central Institute for Labour Protection—National Research Institute, Łódź 90-133, Poland; kamaj@ciop.lodz.pl; 2 Institute of Fermentation Technology and Microbiology, Lodz University of Technology, Łódź 90-924, Poland; justyna.szulc@p.lodz.pl (J.S.); beata.gutarowska@p.lodz.pl (B.G.); 3 Faculty of Chemistry, Adam Mickiewicz University in Poznań, Poznań 61-614, Poland; brycki@amu.edu.pl

**Keywords:** respiratory protective devices, melt-blown nonwovens, antimicrobial textiles with time-dependent biocidal structures, gemini surfactants

## Abstract

Previous studies on nonwovens used for respiratory protective devices (RPDs) were related to equipment intended for short-term use. There is only limited research on the development of biocidal nonwoven fabrics for reusable RPDs that could be used safely in an industrial work environment where there is a risk of microbial growth. Moreover, a new group of biocides with high antimicrobial activity—gemini surfactants, has never been explored for textile’s application in previous studies. The aim of this study was to develop high-efficiency melt-blown nonwovens containing gemini surfactants with time-dependent biocidal activity, and to validate their antimicrobial properties under conditions simulating their use at a plant biomass-processing unit. A set of porous biocidal structures (SPBS) was prepared and applied to the melt-blown polypropylene (PP) nonwovens. The biocidal properties of the structures were triggered by humidity and had different activation rates. Scanning electron microscopy was used to undertake structural studies of the modified PP/SPBS nonwovens. In addition, simulation of plant biomass dust deposition on the nonwovens was performed. The biocidal activity of PP/SPBS nonwovens was evaluated following incubation with *Escherichia coli* and *Aspergillus niger* from the American Type Culture Collection, and with *Pseudomonas fluorescens* and *Penicillium chrysogenum* isolated from the biomass. PP/SPBS nonwovens exhibited antimicrobial activity to varying levels. Higher antimicrobial activity was noted for bacteria (R = 87.85–97.46%) and lower for moulds (R = 80.11–94.53%).

## 1. Introduction

Epidemiologically, airborne dust is the most common way through which harmful biological agents spread in the work environment. Biological particles (microbial cells, toxins) transmitted via these routes can penetrate the human body through the respiratory tract, conjunctivae, nasopharynx mucosa and skin. Respiratory protective devices (RPDs), utilizing particle filtration systems, are required to protect the respiratory tract of workers exposed to harmful bioaerosols. Nonwoven filters, which are the basic material used in RPDs, must have a high capacity to capture fine particles dispersed in the air and be able to provide antimicrobial activity throughout the lifetime of the equipment [1,2,3,4,5]. To achieve these requirements, research has focused on the development of new methods for the functionalization of nonwovens, particularly, related to their biocidal activity [6]. The most common method to confer biocidal activity on natural or synthetic polymers is by chemical modification or by applying a thin-layer of coating. Sureshkumar et al. used silver nanoparticles to endow filtration surfaces with biocidal properties [7]. The authors used a two-step process consisting of (i) applying an adhesive and reductive polydopamine coating on substrates, such as polyethylene, glass, poly(methyl methacrylate), and poly(lactic-*co*-glycolic acid); and (ii) forming a 50–70 nm thick silver nanoparticle layer on the substrates by immersion in silver nitrate solution. Dastjerdi and Shahsavan described a way of combining the actions of silver nanoparticles and titanium dioxide (TiO_2_) in textiles [8]. Dastjerdi and Montazer discussed biocidal agents such as, metallic (TiC) and non-metallic TiO nanoparticles, nanocomposites, titanium nanotubes (TNTs), silver, gold and zinc oxide nanoparticles, copper nanotubes, carbon nanotubes (CNTs), nano-clay and their modified forms [9].

Another method for obtaining nonwovens with biocidal properties was described by Lin et al. [10]. Here, nonwoven fabric made from poly(ethylene terephthalate) (PET) was given biocidal properties by immobilizing two biological antimicrobial agents, ε-polylysine and natamycin, using soft methacrylate nonwoven fabric adhesives. Antimicrobial textile materials were also produced by applying sol-gel coatings with embedded biocidal silver compounds [11]. Novel biocidal polypropylene fabrics have also been synthesized by plasma-assisted graft copolymerization [12]. Nithya et al. studied the synergetic effect of low temperature plasma and enzyme treatments on the physico-chemical properties of cotton fabrics [13]. Yang et al. showed 100% biocidal efficiency against *S. aureus* and 98% efficiency against *E. coli* of modified PET nonwovens [14]. Here, PET nonwovens were pretreated with low-temperature argon plasma, which was subsequently graft polymerized with a water-soluble acrylamide monomer and itaconic acid in order to increase hydrophilicity.

Numerous studies have concentrated on developing nonwovens for RPDs (such as filters and filtering half masks) [15,16,17,18,19,20,21,22,23,24]. Majchrzycka et al. used quaternary ammonium salts embedded in two aluminium-silicate materials (bentonite) and volcanic glass (perlite) to modify melt-blown nonwovens. The addition of perlite increased the hydrophilicity of polypropylene (PP) nonwovens, which proved to be a feature that enhances biocidal activity [15].

The use of gemini surfactant (GS) compounds provides new possibilities for developing bioactive nonwovens for the production of RPDs. GS (otherwise twin or dimer surfactants) are made up of two identical single chain monomers linked together by a linker placed between the hydrophilic groups of the monomers. There are two hydrocarbon chains and two hydrophilic groups in the molecule. GSs possess unique physico-chemical properties. Their critical micelle concentration (CMC) is lower, and they are more efficient at reducing surface tension compared to the conventional monomeric molecules of which they are composed [25,26]. Furthermore, the biocidal activity of the dimeric surfactant is a dozen times higher than its monomeric analogues [27,28].

Based on this, it was assumed that the application of GS, e.g., double quaternary ammonium salts, would help reduce the concentration of biocides required for the functionalization of filtering nonwovens, which would significantly increase the safety of RPDs use. Furthermore, it was assumed that temporal release of these agents from the fibre surface of the filtering materials would reduce user contact with the biocide during regular RPDs use at workstations.

So far, studies on the development of a biocidal nonwoven fabric for reusable RPDs that can be used safely for several days in industrial environments are lacking. Therefore, the purpose of the study was two-fold: (i) to devise high efficiency filtering nonwovens containing GS-based biocides with time-dependent biocidal activity for the construction of RPDs for long-term use; and (ii) to validate the effectiveness of the antimicrobial activity under conditions simulating their use in plant biomass processing workstations. To ensure the assumed functions of the filtering material a set of porous biocidal structures (SPBS), consisting of five individual types of structures (HA-2LG1 to HA-10L) obtained by deposition of various amounts of hexamethylene-1,6-bis(*N*,*N*-dimethyl-*N*-dodecyldodecylammonium bromide) (GS-12-6-12) on halloysite nanocrystals, was applied to the melt-blown nonwovens during their production. The principle of biocidal agent action assumed that it would be temporally activated in the halloysite carrier incorporated into polymeric fibers during repeated use of the equipment at the workstation. The differentiation of the activation time was ensures by the addition of various amounts of 1,2-propanediol that regulated the amount of moisture adsorbed by the structures from the exhaled air.

## 2. Results and Discussion

### 2.1. Evaluation of the Morphology of the Nonwovens

**Figure 1:**
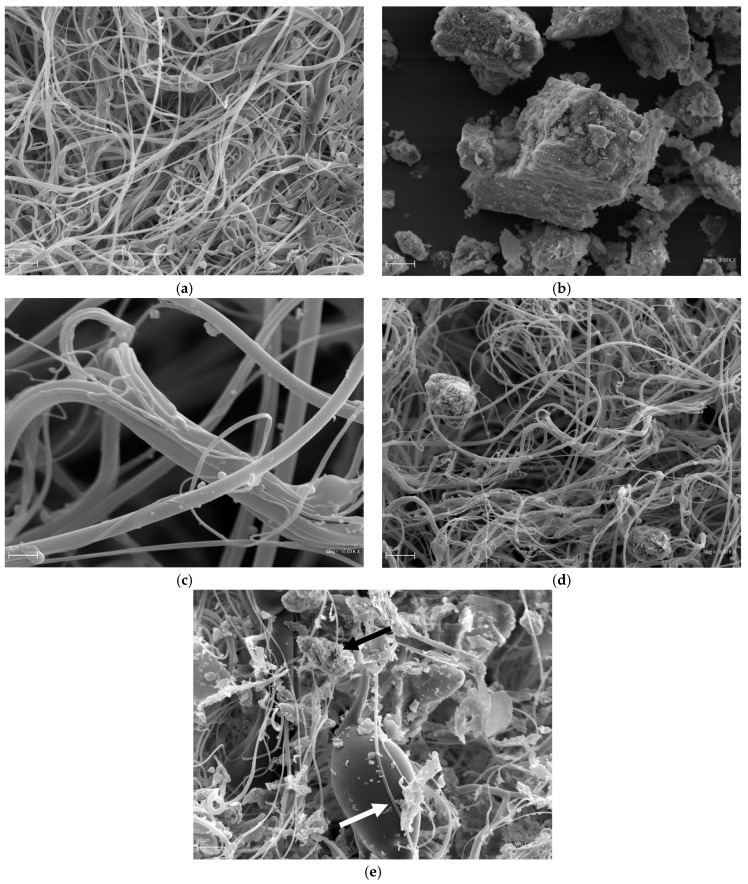
SEM images of: (**a**) control PP nonwoven (mag. 3000×); (**b**) SPBS particles and agglomerates (mag. 3000×); (**c**) PP/SPBS nonwovens with SPBS grains melted into PP fibres (mag. 15,000×); (**d**) PP/SPBS nonwovens with SPBS agglomerates entangled within the fibres (mag. 3000×); (**e**) PP/SPBS nonwovens with deposited biomass dust particles (SPBS agglomerate—black arrow, dust particle—white arrow, mag. 3000×).

Scanning electron microscopy (SEM) was employed to investigate the morphologies of the PP/SPBS nonwovens before (Figure 1c,d) and after biomass dust deposition (Figure 1e). For comparison, the SEM images of pristine PP nonwovens (Figure 1a) and SPBS particles (Figure 1b) were also obtained.

The average diameter of the pristine PP nonwoven fibres (Figure 1a) was 0.7 ± 0.6 µm. For PP/SPBS nonwovens (Figure 1c), fibres of similar diameter of 0.9 ± 1.3 µm were obtained. In contrast, the average size of the SPBS granule (Figure 1b) was 19.7 ± 27.3 µm, which was on average over 20-times larger than the average diameter of the PP and PP/SPBS fibres (*p* < 0.05).

The granules in the nonwoven structure were attached in two ways. Smaller grains, of dimensions ranging from 0.4 to 2 µm, were fixed on the surface of the fibres (Figure 1c), whilst agglomerates of SPBS grains of dimensions from 2 to 180 µm were embedded in-between entangled fibres and were simultaneously fixed to several surrounding fibres (Figure 1d). Here we must mention that the technique of producing nonwovens in which SPBS were added to a semiliquid polymer at the stage of fibre formation, involved partially melting them into the PP material [15,19,29,30]. The described effect was previously confirmed in SEM studies and by analysing the content of biologically active substances in the modified PLA fibres using elementary and UV spectrophotometric analyses. The method for introducing a biocidal modifier was described in a patent [30].

The criterion for selecting a method for the functionalization of melt-blown nonwovens was their expected use in the construction of RPDs. Therefore, it was necessary to account for the fact that filtering nonwovens should provide high filtration efficiency along with a low degree of air flow resistance. For this reason, finishing technologies that reduce the porosity of the nonwoven surface layer, as a result of applying an even thin layer with biocidal properties, had limited use. The application of chemical modification of the fibre surface constituted a problem for the safety of the equipment in the anticipated conditions of the polluted work environment. Based on this, it was important for the biocidal agent to be permanently bonded to the fibre. Moreover, the use of GS as an active agent ensured that only limited quantities of it remained in the final product. The incorporation of the biocidal modifier into the structure of PP fibres provided durability and hence higher resistance to the working conditions. Based on these criteria, the direct formation of fibres from the polymer melt was a good solution [15,19,31]. Indeed, in this process, it is possible to introduce functional additives at the stage of fibre formation.

Figure 1e shows PP/SPBS after the process of loading the nonwoven with biomass dust. The distribution of the dust agglomerates in the nonwoven was homogenous. Moreover, no areas of higher density of biomass were found on the PP/SPBS structure. Similar distribution was observed under conditions of RPDs use in previous studies [32]. This is proof that our laboratory simulation of pollution gave a result identical to that of RPDs use at industrial workstations.

### 2.2. Survival of Microorganisms on Filter Materials

**Figure 2:**
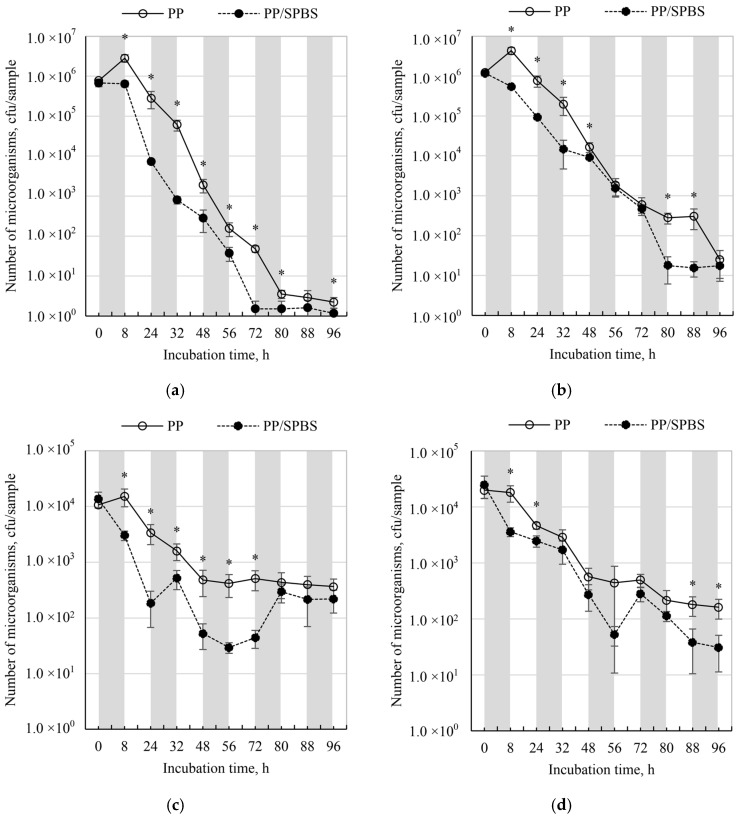
Number of microorganisms on control and bioactive nonwovens during incubation simulating 5 days of use in plant biomass processing workstations: (**a**) *E. coli*; (**b**) *P. fluorescens*; (**c**) *A. niger*; (**d**) *P. chrysogenum*. *—Significant differences between the number of microorganisms on the PP and PP/SPBS nonwovens (one-way ANOVA, *p* < 0.05; Tukey’s test, *p* < 0.05).

The number of microorganisms on both melt-blown nonwovens (PP and PP/SPBS) over a cycle simulating five days of RPDs use (8 h work shift and 16 h break) in a work environment is shown in Figure 2a–d.

The number of *E. coli* bacteria on PP nonwoven ranged from 2.22 × 10^0^ cfu/sample (following 96 h incubation) to 2.83 × 10^6^ cfu/sample (following 8 h incubation). On the PP/SPBS nonwoven *E. coli* numbers ranged from 1.17 × 10^0^ cfu/sample (after 96 h incubation) to 6.82 × 10^5^ cfu/sample (t = 0) (Figure 2a). On PP nonwoven, after the first 8 h of incubation in enhanced humidity, an increase in *E. coli* numbers was observed (survival index, N = 364.81%). Subsequently, the number of bacteria on both nonwovens (PP and PP/SPBS) diminished with incubation time. A faster rate of bacterial death was detected on PP/SPBS nonwoven—*E. coli* survival index was close to N = 0% after just 32 h, while it took 48 h for the control nonwoven.

The number of *P. fluorescens* bacteria on PP nonwoven reduced from 4.33 × 10^6^ cfu/sample (after 8 h incubation) to 2.48 × 10^1^ cfu/sample (after 96 h incubation) (Figure 2b). On PP/SPBS nonwoven, *P. fluorescens* number reduced from 1.18 × 10^6^ cfu/sample (t = 0) to 1.57 × 10^1^ cfu/sample (after 88 h incubation). On the PP nonwoven, after the first 8 h of incubation in enhanced humidity, similar to *E. coli*, an increase in *P. fluorescens* numbers was seen (survival index, N = 353.26%) (Table 1). Over subsequent hours of incubation, bacteria survival index decreased reaching almost 0% after 56 h and 48 h for PP and PP/SPBS, respectively.

*A. niger* number on PP nonwoven reduced from 1.52 × 10^4^ cfu/sample (after 8 h incubation) to 3.67 × 10^2^ cfu/sample (after 96 h incubation). On PP/SPBS nonwoven, *A. niger* number reduced from 1.37 × 10^4^ cfu/sample (t = 0) to 2.15 × 10^2^ cfu/sample (following 88 h incubation) (Figure 2c). On PP nonwoven, following the first 8 h of incubation in enhanced humidity conditions, an increase in the *A. niger* number (survival index, N = 142.19%) was observed, as was found in the case of bacteria. Over subsequent hours of incubation, mould survival index on PP/SPBS and PP nonwoven decreased, reaching N = 3.44–4.52% after 48–96 h. The survival of mould on PP/SPBS was lower and amounted to a maximum, N = 22% after 8 h of incubation, further decreasing to N = 0.22–3.78% after prolonged incubation (Table 1).

The number of *P. chrysogenum* mould on PP nonwovens decreased from 2.0 × 10^4^ cfu/sample (t = 0) to1.62 × 10^2^ cfu/sample (following 96 h incubation). On PP/SPBS nonwoven *P. chrysogenum* number decreased from 2.48 × 10^4^ cfu/sample (t = 0) to 3.10 × 10^1^ cfu/sample (following 96 h incubation) (Figure 2d). On both PP and PP/SPBS, *P. chrysogenum* mould had very low viability as evidenced by a survival index, N = 0.81–90% and N = 0.12–14.43% for PP and PP/SPBS, respectively (Table 1). There was a reduction in bacterial numbers from R = 3.80 to 97.46% depending on the species and incubation time. Higher antimicrobial activity was recorded for *E. coli* bacteria obtained from pure cultures than for *P. fluorescens* isolated from the work environment. As the incubation duration increased, there was a greater rate of reduction in bacterial numbers: from R = 12.23 to 97.46% for *E. coli* and from R = 3.80% to 87.85% for *P. fluorescens*. The reduction in mould numbers was ascertained only after 24 h and 32 h incubation, and ranged from R = 80.09 to 94.46% depending on the species. The ATCC *A. niger* strain had higher sensitivity to PP/SPBS nonwoven (R = 80.11–94.53%), while the *P. chrysogenum* environmental isolate had lower sensitivity (R = 46.78–80.09%) (see Table 1). Similar observations, i.e., lower sensitivity to GS-12-6-12, of bacteria and fungi isolated from the environment compared to collection strains of the same species, were made in earlier studies that determined minimal inhibitory concentrations (MIC) [27].

The maximum reduction in microorganism numbers was seen after 24 h or 32 h of incubation depending on the species. This indicates that PP/SPBS antimicrobial activity may vary depending on the type of microorganism and its growth phase. Brycki et al. found that the MIC for 48 h mycelium development with GS-12-6-12 was significantly higher than that for conidia, which may be due to the characteristics of mycelium growth [25].

Dust-fed PP nonwoven constituted an environment favourable for the growth of bacteria and moulds used in the study at 24 h of incubation time (survival index N = 90–364%), following which microorganisms died (N = 0.02–4.52%). The death of microorganisms was probably caused by temporary storage in low relative humidity of the air (RH = 45%)—simulating the break in RPDs usage by a worker. This indicates that standard RPDs should be stored in conditions of low humidity (RH = 45%) as such conditions impede the growth of microorganisms on filter material. This conclusion is in agreement with previous publications. Pasanen et al. conducted studies on filters made up of different proportions of glass and cellulose fibers [1]. The efficiency of filtration and microbial growth in filtering materials during storage conditions were determined. It was ascertained that filters with a greater content of hygroscopic cellulose were more prone to microorganism growth under conditions of increased humidity. In PP nonwovens, fibre surface evaporation is very quick, resulting in fast drying of filters. However, taking into account the fact that during RPDs use there is an increase in its humidity [33,34], subsequent use of the RPDs containing PP nonwoven filters would increase microorganism growth on the filter. This phenomenon was not observed in the case of PP/SPBS. 

PP/SPBS nonwovens exhibited varying antimicrobial activity depending on microorganism species and incubation times. Higher antimicrobial activity of PP/SPBS nonwoven was found for bacteria (up to R = 87.85–97.46%) and lower for moulds (max. R = 80.11–94.53%). Higher antimicrobial activity of PP/SPBS was seen for bacteria and mould strains originating from pure culture collections than those isolated from plant biomass processing work environment. Antimicrobial activity of PP/SPBS varied over time and depended on microorganism species. Maximal microorganism numbers were seen after 24 h or 32 h of incubation depending on the microorganism species. This was followed by a rapid reduction of microorganism survival (near 0%), which shows that the concentration of GS-12-6-12 that was selected for the fibres was correct. Previous research on GS-12-6-12 antimicrobial activity was performed using two-fold serial dilutions [25] on: *Staphylococcus aureus, Pseudomonas aeruginosa, Candida albicans, Aspergillus niger, Penicillium chrysogenum* microorganisms of various origins. GS-12-6-12 concentrations that inhibited the growth of 24 h cultures of these microorganisms from 0.0036 µM/mL to 0.008 µM/mL (0.0002–0.008%, respectively) were established [25,27]. For fungal cultures, the authors noted 3 to 21 times higher value of MIC by increasing culture time from 24 to 48 h.

Gutarowska et al. stressed that it is not possible, based on MIC values for dyes and finishing agents, to draw conclusions about what quantities of biocidal substances in fabrics will be sufficient to impart adequate or expected antimicrobial properties to those fabrics [17]. The concentration of bioactive substances in nonwovens should be higher than their MIC values because of impaired contact of biocide with microorganisms within the nonwoven compared to their liquid culture. The choice of biocide concentration is a challenging task and should be carried out individually for each biocide and fabric [17].

The GS-12-6-12 concentrations used in the current study were only 1.6-times higher than that determined by Brycki et al. for 48 h fungal cultures [25]. A similar reduction in microorganism numbers, as in this study, was also obtained by Majchrzycka et al. when using quaternary ammonium salts set in perlite at significantly higher concentrations (10, 15, 20% of bioperlite in proportion to PP mass) for the modification of melt-blown PP nonwovens [15]. The reduction of the amount of the active agent, in relation to the previously described biocidal nonwovens, was possible thanks to the use of SPBS containing GS-12-6-12 with much higher biocidal activity compared to the conventional monomeric molecules. This is in agreement with the premise of minimising biocide content in filtering nonwovens used in RPDs and it has important safety implication for future users of bioactive RPDs due to the plausible adverse health effects of excessive and long-term biocide use [35].

Moreover, in case of newly developed biocidal structures users’ contact with the active substance is largely reduced as a result of temporal activation of biocide in the filtering materials. In contrast to the previously used biocides, SPBS contains of a mixture of individual halloysite composites with varying content of active agent (GS-12-6-12) and the substance controlling the capacity of halloysite to absorb water from exhaled air (1,2-propanediol). The active agent is inactive in the anhydrous state and introducing 1,2-propanediol to the supramolecular system to provide enhanced hydrophilicity of the structures ensures the onset of its biocidal action. Thus the variation in the amount of 1,2-propanediol ensures differentiation of the activation time of structures. The greater the amount of 1,2-propanediol in the composition of a given structure is, the faster the wetting of their surface and the activation of the active substance occurs. This progressive time-dependent activation of biocidal agent in SPBS ensures that the active substance is able to react with the users’ skin for shorter periods of RPDs use, which may contribute to their safety.

## 3. Materials and Methods

### 3.1. Nonwovens with Time-Dependent Biocidal Structures

Moplen HP456J isotactic PP (MFI 3.4 g/10 min at 230 °C/2.16 kg) purchased from Basell Orlen Polyolefins (Płock, Poland) was used for the production of melt-blown nonwovens. Antimicrobial properties were obtained by applying a set of porous biocidal structures (SPBS) to the melt-blown nonwovens. The method for obtaining SPBS was described in detail by Majchrzycka et al. [36,37].

**Figure 3:**
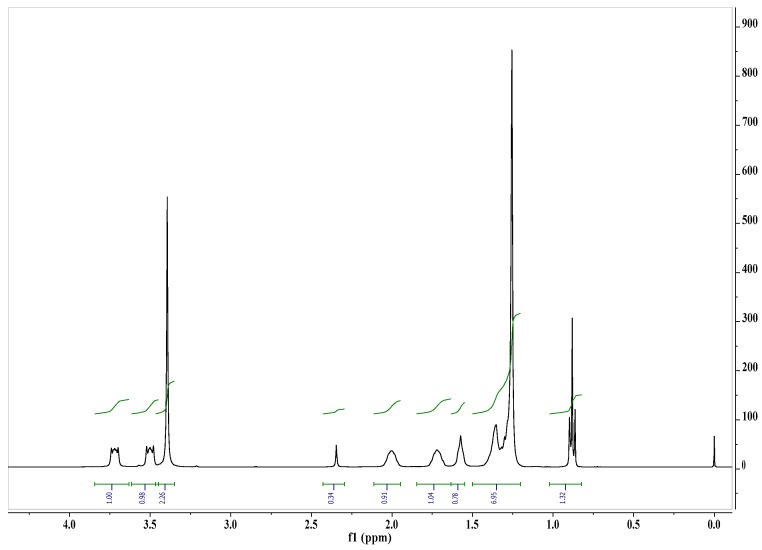
^1^H-NMR spectrum of hexamethylene-1,6-bis(*N*,*N*-dimethyl-*N*-dodecylammonium bromide) in CDCl_3_.

**Figure 4:**
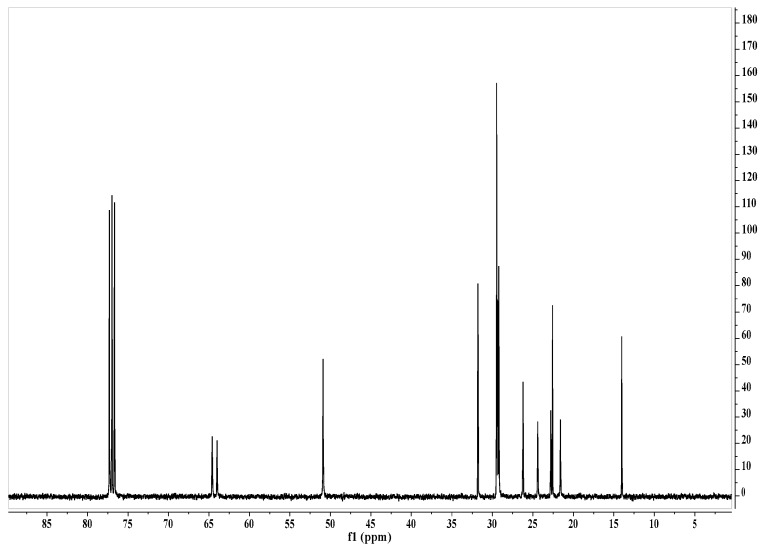
^13^C-NMR spectrum of hexamethylene-1,6-bis(*N*,*N*-dimethyl-*N*-dodecylammonium bromide) in CDCl_3_.

Hexamethylene-1,6-bis(*N*,*N*-dimethyl-*N*-dodecylammonium bromide) (GS-12-6-12) was prepared by reaction of *N*,*N*-dimethyl-*N*-dodecylamine (36.4 g, 0.18 M) (Aldrich, Munich, Germany) with 1,6-dibromohexane (21.4 g, 0.08 M) (Aldrich) in acetonitrile (120 mL) under reflux for 6 h, according to a procedure described in literature [25]. The crude product was crystallized from acetonitrile to give white crystals of GS-12-6-12 (Yield 90.4%, m.p. 231–232 °C; elemental analysis: found (calc.) %C 60.51 (60.88); %H 11.65 (11.12); %N 4.09 (4.18); ES + MS *m*/*z* 255 (C34H74N2/2). The purity of the synthesized compound was confirmed by ^1^H-NMR (Figure 3) and ^13^C-NMR (Figure 4).

Biocidal structures were obtained by deposition of GS-12-6-12 on nanocrystals of halloysite. The dry form of the above structures shows no antibacterial activity. To trigger antibacterial activity a water stimulus is essential. To enhance the capacity of halloysite composites to absorb water from exhaled air, 1,2-propanediol was introduced in the structures.

The content of the active substance was selected according to the projected activity time of a given type of structure. SPBS were obtained by mixing in equal quantities (100 g each) five types of structures with varying content of GS-12-6-12 and 1,2-propanediol. The characteristics of the structures are shown in Table 2.

### 3.2. Manufacturing of Melt-Blown Nonwovens

Melt-blown technique was used for the preparation of nonwovens with SPBS. Experimental work was conducted using a single-screw laboratory extruder. The temperatures of the heating zones of the extruder ranged from 270 °C to 280 °C, and the polymer melt was blown with hot air stream at a temperature of 270 °C. SPBS, at a concentration of 7%, was added, using a specially constructed pneumatic nozzle located in the fibre-forming head channel, directly into the fibre-forming zone, bypassing the high temperature zone [15,19,30]. After leaving the nozzle, SPBS particles hit the stream of semi-liquid PP fibres so that they could be permanently fused to the polymeric material. PP nonwovens with SPBS (PP/SPBS) with an average surface mass of 56.7 ± 1.6 g/m^2^ and average thickness of 1.4 ± 0.1 mm, were obtained. The final concentration of GS in the nonwoven amounted to 0.336%. In addition, pristine PP nonwovens of similar surface mass and thickness (55.4 ± 1.3 g/m^2^; 1.3 ± 0.1 mm) were fabricated to act as a control variant. Paraffin oil aerosol penetration index and air flow resistance values were determined for the nonwovens according to EN 13274-7:2008 and EN 13274-3:2001 standards [38,39]. They equalled 0.7% and 300 Pa, and 1.2% and 290 Pa for PP/SPBS and pristine PP (control) nonwovens, respectively.

### 3.3. Simulation of Use in Organic Dust Pollution Conditions

In order to simulate the deposition of dust in the nonwoven fabric during RPDs use, the PP/SPBS biocidal nonwoven samples and PP control nonwovens were loaded with organic dust obtained from an electric heat and power station processing plant biomass [5] at a laboratory workstation equipped with a dust chamber, rotary dust feeder and sample holder [32]. Sample preparation consisted of placing the nonwovens in a holder, in the upper part of the chamber. Next, grounded, dried and sterile biomass dust was passed from the rotary dust feeder through the sample at a constant volume flow rate of 90 L/min. The experiments were conducted at a temperature of 23.2 °C and relative humidity of 42%. The dust feeding time was selected such that the mass of dust deposited matched the amount deposited on filter layers of RPDs during use. As a result, a dust deposition level of 3% of the nonwoven fabric mass was obtained.

### 3.4. Morphology of Filtering Nonwovens

The experiments on PP/SPBS bioactive and control PP nonwovens were done both before and after organic dust was applied. The morphological characteristics of the selected nonwovens were analysed using scanning electron microscopy (SEM, Carl Zeiss SMT Ltd., Cambridge, UK). In particular, the fibre and SPBS size range, the uniformity of SPBS distribution in the biocidal nonwoven fabric and the way in which SPBS particles were attached to the fibres were evaluated. In addition, the distribution of organic dust within PP/SPBS nonwoven samples following dust application was assessed.

### 3.5. Microorganisms Tested

The degree of microbial survival was determined using microorganisms from the American Type Cell Collection (ATCC): *Escherichia coli* (ATCC 10536) and *Aspergillus niger* (ATCC 16404). In addition, analysis was also carried out using microorganisms isolated from the plant biomass processed at workstations with a high concentration of organic dust (*Pseudomonas fluorescens* and *Penicillium chrysogenum* [5]). The criterion for selecting environmental isolates was their high frequency of isolation from biomass samples as well as their potential harmfulness to the health of workers [40].

The microorganisms were stored on agar slants: bacteria on TSA (Tryptic Soy Agar, Merck, Darmstadt, Germany), and moulds on MEA (Malt Extract Agar, Merck) at a temperature of 4 °C. Bacteria were activated in TSB medium (Tryptic Soy Broth, Merck) at a temperature of 30 °C for 24 h and moulds on MEA slants at 27 °C for 5 days.

A microorganism inoculum was prepared. Bacteria colonies were transferred into10 mL of TSB medium and incubated in conditions as described above. In the case of moulds, colonies from MEA slants were washed using 10 mL distilled water with 0.01% of Tween^®^ 80. The number of microorganisms in the inoculum (between 2.0 × 10^8^–5.5 × 10^9^ cfu/mL) was determined using a Thom cell counting chamber and the plate count method. Verifying the density of the inoculum in the Thom chamber indicated that in the inoculum approximately 99% of all cells of moulds constituted to spores.

### 3.6. Survival of Microorganisms on Filtering Nonwovens

Nonwoven samples of an area of 4 cm^2^ (squares 2 × 2 cm; average mass of samples was 0.0125 g) were inoculated with 10 µL of microbial inoculum and 15 µL sterile saline solution (0.85% NaCl) to obtain 200% initial mass humidity. Inoculum as well as sterile saline solution was applied homogenously onto the samples using a pipette with sterile tips for the distribution of small water droplets over the whole sample. The samples inoculated with microorganisms were placed in sterile Petri plates and incubated in conditions simulating the use of RPDs. For this purpose, the samples were initially incubated for 8 h (half-mask exploitation time equivalent to a single work shift) in a climatic chamber (T = 28 ± 2 °C; RH = 80%). Thereafter, the samples were incubated at room temperature (T = 24 °C; RH = 45%) for 16 h (16 h corresponded to storage time during the break, after use). The cycle described above was repeated 5 times (simulating 5 days of use).

Antimicrobial efficiency of the melt-blown nonwovens was determined using the AATCC 100 quantitative method [41]. Samples for the analysis were collected immediately after seeding the microorganisms (t = 0), and after 8, 24, 32, 48, 56, 72, 80, 88 and 96 h of incubation. Following the appropriate incubation time, the nonwoven samples were transferred to sterile containers containing 50 mL of 0.85% NaCl saline solution and shaken for 5 min. Next, serial dilutions of samples (from 10^−1^ to 10^−6^ in duplicate) were made in 0.85% NaCl saline solution and seeded onto sterile Petri dishes flooding them with semi-solid TSA (Tryptic Soy Agar, Merck) medium for bacteria and MEA (Malt Extract Agar, Merck) for moulds. Plates were incubated at 37 ± 2 °C for 24 h (bacteria) or 27 ± 2 °C for 72 h (moulds). Subsequently, the colonies were counted (the results were expressed as cfu/sample). The experiments were performed independently three times for each nonwoven tested.

### 3.7. Mathematical Analysis

Microbial survival index (N) for both melt-blow nonwovens (PP and PP/SPBS) was calculated according to the formula:

(1) $$N=\frac{N_{t}}{N_{0}}\cdot 100\%$$

where $N_{0}$ is the number of microorganisms present on melt-blown nonwoven at time t = 0 h (cfu/sample) and $N_{t}$ is the number of microorganisms present on melt-blown nonwoven after a specified incubation time (t = 8, 24, 32, 48, 56, 72, 80, 88, 96 h) (cfu/sample).

The reduction in the number of microorganism (R) following contact with PP/SPBS nonwoven was calculated using the formula:

(2) $$R=\frac{K-B}{K}\cdot 100\%$$

where K is the number of microorganisms present on PP melt-blown nonwovens after subsequent hours of incubation (cfu/sample) and B is the number of microorganisms present on PP/SPBS melt-blown nonwovens after subsequent hours of incubation (cfu/sample).

The reduction in the number of microorganisms was calculated for 8, 24 and 32 h of incubation. It was not evaluated for longer incubation times due to low survival rate of microorganisms, which did not exceed 4% (the number of microorganisms on PP/SPBS did not exceed the level of 10^3^).

The arithmetic mean and standard deviation for the number of microorganisms on the surface of the tested materials were calculated. Differences between the number of microorganisms after a particular incubation time and those from the control samples were analysed using one-way analysis of variance (ANOVA). Differences were considered significant at *p* < 0.05. All data were analysed using the Origin 6.1 statistical software program.

## 4. Conclusions

The study describes a method for obtaining high efficiency filtering nonwovens endowed with biocidal properties that is intended for the construction of RPDs for long-term use. Their effectiveness was validated in conditions simulating their use at a plant biomass processing workplace.

Nonwovens with high protective characteristics of aerosol filtration (penetration at the level of 0.7%, air flow resistance at the level of 300 Pa), showing antimicrobial action at differential levels, were obtained. Higher antimicrobial activity was noted for bacteria (max. R = 87.85–97.46%) and lower for moulds (max. R = 80.11–94.53%).

On the basis of the results of this study, it was concluded that the use of PP/SPBS filtering nonwovens with biocidal action is suitable for the production of re-usable RPDs. The reason for this is that regardless of microclimatic conditions during work and storage of the equipment, hygienic conditions can be maintained without employee intervention. This will ensure conditions for safe use of the equipment in the work environment, where employees are exposed to harmful aerosols.

Finally, when using biocide-impregnated nonwovens in RPDs, it is important to not only reduce the amount of biocidal agent, but also activate it gradually over time. This is essential to improve safety, but maintain efficacy, which we address in our study.

## Figures and Tables

**Table 1:** Reduction in the number of microorganisms and their survival on PP/SPBS nonwoven.

Microorganisms	Reduction of Microorganisms Number, R (%)			Survival Index, N (%)		
	8 h	24 h	32 h	8 h	24 h	32 h
*Escherichia coli*	12.23	77.15	97.46	94.96	1.06	0.12
*Pseudomonas fluorescens*	3.80	87.50	87.85	45.90	7.82	1.24
*Aspergillus niger*	- ^1^	80.11	94.53	22.07	1.35	3.78
*Penicillium chrysogenum*	- ^1^	80.09	46.78	14.43	9.93	6.85

^1^ No reduction was observed.

**Table 2:** The characteristics of the structures used to prepare SPBS.

No.	Type of Biocidal Structures	Concentration of GS-12-6-12, %	Concentration of 1,2-Propanediol, %
**1**	HA-2L G5	2	5
**2**	HA-5L G4	5	4
**3**	HA-5L G3	5	3
**4**	HA-2L G1	2	1
**5**	HA-10L	10	0
